# Involvement of the OTUB1‐YAP1 axis in driving malignant behaviors of head and neck squamous cell carcinoma

**DOI:** 10.1002/cam4.6735

**Published:** 2023-11-20

**Authors:** Shengjian Jin, Takaaki Tsunematsu, Taigo Horiguchi, Yasuhiro Mouri, Wenhua Shao, Keiko Miyoshi, Hiroko Hagita, Motoharu Sarubo, Natsumi Fujiwara, Guangying Qi, Naozumi Ishimaru, Yasusei Kudo

**Affiliations:** ^1^ Department of Oral Bioscience Tokushima University Graduate School of Biomedical Sciences Tokushima Japan; ^2^ Department of Oral Molecular Pathology Tokushima University Graduate School of Biomedical Sciences Tokushima Japan; ^3^ Department of Oral Healthcare Management Tokushima University Graduate School of Biomedical Sciences Tokushima Japan; ^4^ Guangxi Key Laboratory of Tumor Immunology and Microenvironmental Regulation Guilin Medical University Guilin China

**Keywords:** deubiquitinating enzyme (DUB), head and neck squamous cell carcinoma, invasion, OTUB1, YAP

## Abstract

**Background:**

Comprehending the molecular mechanisms underlying head and neck squamous cell carcinoma (HNSCC) is vital for the development of effective treatment strategies. Deubiquitinating enzymes (DUBs), which regulate ubiquitin‐dependent pathways, are potential targets for cancer therapy because of their structural advantages. Here we aimed to identify a potential target for HNSCC treatment among DUBs.

**Methods:**

A screening process was conducted using RNA sequencing data and clinical information from HNSCC patients in the TCGA database. A panel of 88 DUBs was analyzed to identify those associated with poor prognosis. Subsequently, HNSCC cells were modified to overexpress specific DUBs, and their effects on cell proliferation and invasion were evaluated. In vivo experiments were performed to validate the findings.

**Results:**

In HNSCC patients, USP10, USP14, OTUB1, and STAMBP among the screened DUBs were associated with a poor prognosis. Among them, OTUB1 showed the most aggressive characteristics in both in vitro and in vivo experiments. Additionally, OTUB1 regulated the stability and nuclear localization of YAP1, a substrate involved in cell proliferation and invasion. Notably, OTUB1 expression exhibited a positive correlation with the HNSCC‐YAP score in HNSCC cells.

**Conclusions:**

This study highlights the critical role of OTUB1 in HNSCC progression via modulating YAP1. Targeting the OTUB1‐YAP1 axis holds promise as a potential therapeutic strategy for HNSCC treatment.

## Graphical abstract

This study identified OTUB1 as a critical deubiquitinating enzyme (DUB) strongly associated with unfavorable prognoses in head and neck squamous cell carcinoma (HNSCC) patients. Through in vitro and in vivo experiments, it was demonstrated that OTUB1 plays a significant role in promoting HNSCC progression by modulating the stability and nuclear localization of Yes‐associated protein 1 (YAP1). Targeting the OTUB1‐YAP1 axis holds promise for the development of novel therapeutic strategies against HNSCC.

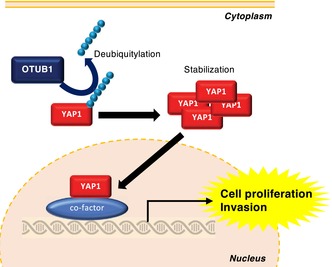

## 1 INTRODUCTION

Head and neck squamous cell carcinoma (HNSCC) encompasses an array of malignant tumors originating from the upper respiratory and digestive tracts, specifically the oral cavity, pharynx, and larynx.^1^, ^2^ Despite advancements in early detection and treatment modalities, the prognosis for HNSCC patients remains unimproved. In recent years, there have been significant advancements in cancer drug therapy, including targeted treatments like epidermal growth factor receptor inhibitors and immune checkpoint inhibitors for HNSCC.^3^ However, the options for molecular‐targeted drugs in HNSCC are limited compared to other cancers. Thus, there is an urgent need to develop new drugs that target the specific molecular processes driving tumor growth and development.

Ubiquitination and deubiquitination are two popular ways of post‐translational modification of proteins and affect intracellular localization, stability, and function of target proteins. Deubiquitinating enzyme (DUB) maintains the dynamic balance of the ubiquitin system by cleaving the ubiquitin polymer chain or completely removing the ubiquitin chain from the ubiquitin protein.^4^ To date, approximately 100 DUBs have been identified within six distinct families: UBP/USP, UCH, JAMM, OTU, MJD, and MCPIP.^4^, ^5^, ^6^ DUBs play a pivotal role in the regulation of ubiquitin‐dependent pathways including histone modification, cell cycle regulation, cell differentiation, apoptosis, endocytosis, autophagy, and DNA repair via protein degradation, protein function, gene expression, and signal transduction.^4^, ^5^ Cumulative evidence suggests that aberrant regulation of DUBs is implicated in cancer progression.^6^ Although the mechanism and downstream effects governed by DUBs are not fully elucidated, it is particularly crucial to further investigate the intricacies of this regulatory mechanism as a prospective target for HNSCC treatment. Given their structural advantages, DUBs can be regarded as potential therapeutic drug targets for cancer.^7^

Therefore, here we tried to identify DUBs that is involved in malignant behaviors of HNSCC by in silico, in vitro, and in vivo screenings to pinpoint a potential DUB‐based target for addressing HNSCC. Among screened DUBs, we identified OTUB1 (Ovarian Tumor Domain Ubiquitin Aldehyde Binding Protein 1) as a factor exhibiting significant aggressive tendencies in HNSCC. Furthermore, we discovered that the OTUB1‐YAP1 (Yes‐associated protein 1) axis was implicated in the malignant behaviors of HNSCC cells.

## 2 MATERIALS AND METHODS

### 2.1 TCGA data analysis

For survival analysis, the RNA‐seq count values and patient clinical information in TCGA‐HNSC (PanCancer Atlas) dataset were downloaded from the cBioPortal Public Datahub (https://github.com/cBioPortal/datahub). The z scored RSEM normalized counts were used as gene expression values. One patient's data with ‘NA’ in overall survival (OS) time was excluded and the data from 514 cases of HNSC, each with survival information, were used for subsequent analysis. Univariate Cox regression analysis was performed to evaluate the association between the patient's OS and the continuous mRNA expression value of each DUB using the R package *survival*. DUBs with the top 10 and bottom 10 hazard ratios (HR) were selected, and the log10 transformed HR values were visualized as a heatmap using the R package *pheatmap*. The HNSCC patients were divided into two groups based on the median expression levels of USP10, STAMBP, USP14, and OTUB1. Log‐rank test for overall survival between these two groups were performed and the *p* value along with Kaplan–Meier plots were visualized. To identify target proteins of OTUB1, we divided HNSC patients into high and low OTUB1 mRNA expression groups and compared the protein expression between these groups on the cBioPortal website (https://www.cbioportal.org). The result was downloaded from the cBioPortal website and visualized as a volcano plot using the R package *ggplot2*.

### 2.2 Antibodies and reagents

The antibodies used were as follows: anti‐OTUB1 (ab232581, Abcam); anti‐YAP (14074, Cell Signaling Technology); anti‐FLAG (F3165, Sigma); anti‐HDAC1 (#39531, Active Motif); anti‐PD‐L1 (ab205921, Abcam); anti‐Myc‐Tag (#2276, Cell Signaling); anti‐FoxM1 (#20459, Cell Signaling); anti‐Snail (#3879, Cell Signaling); anti‐xCT/SLC7A11 (#98051, Cell Signaling); and anti‐β‐actin (#A5316, SIGMA). MG132 and Cycloheximide (CHX) were purchased from CALBIOCHEM (#474791) and FUJIFILM Wako (#03720991), respectively.

### 2.3 Cell lines

The cell lines HSC2, HSC3, HSC4, HO‐1‐U‐1, CA9‐22, SAS, HO‐1‐N‐1, and HEK293T were obtained from Japanese Collection of Research Bioresources Cell Bank. HOC313, HOC621, HOC719‐PE, and HOC719‐NE cells were provided by Prof. Nobuyuki Kamata (Hiroshima University).^8^ KOSCC25B and KOSCC33A cells were obtained from Prof. Sam‐Pyo Hong (Seoul National University). MSCC1, MSCC1‐inv1, and SpSCC cells were previously established in our laboratory.^9^, ^10^, ^11^ Most cells were maintained in Dulbecco's Modified Eagle Medium (FUJIFILM Wako) supplemented with 10% heat‐inactivated FBS (Nichirei Bioscience Inc.) under conditions of 5% CO_2_ in air at 37°C. MSCC1 and MSCC1‐inv1 cells were maintained in Keratinocyte SFM (Thermo Fisher Scientific) under conditions of 5% CO_2_ in air at 37°C.

For cell proliferation was assessed using a Cell counter (Z Series Coulter Counter, Beckman Coulter). Briefly, 5 × 10^3^ cells were seeded into each well of a 24‐well culture plate. Cells were counted at day 0, 2, 4, and 6.

### 2.4 Quantitative real‐time PCR (qPCR)

Total RNA was isolated from cells using the RNeasy Mini Kit (Qiagen) and purity was determined by a standard spectrophotometric method. From total RNA, cDNA was synthesized by using the PrimeScript RT Master Mix (Takara Bio Inc). mRNA expression was determined by using a CFX connect real‐time system (Roche) with SYBR Premix Ex Taq II reagent (Takara Bio Inc). qPCR was performed as described previously.^12^ The following primer sequences were used. *OTUB1*: forward, 5′‐GTCTGCCAAGAGCAAGGAAG‐3′ and reverse, 5′‐GAGGTCTGCTTCTCCACCTG‐3′; *STAMBP*: forward, 5′‐TTGCAAGCTGCACAAGTACC‐3′ and reverse, 5′‐TCTCTCTGCACCTGGCCTAT‐3′; *USP10*: forward, 5′‐CAGTGACACTTTGCCGAGAA‐3′ and reverse, 5′‐TTCCGCCTCCACATTAGAAC‐3′; *USP14*: forward, 5′‐ATTTCAGGCTTTGCAGGCTA‐3′ and reverse, 5′‐CCCAGCTGTGCTCACTCATA‐3′; *GAPDH*: forward, 5′‐TCCACCACCCTGTTGCTGTA‐3′ and reverse, 5′‐ACCACAGTCCATGCCATCAC‐3′.

### 2.5 Plasmids and siRNA

The FLAG‐OTUB1 pcDNA3.1 were kindly provided by Dr. Mushui Dai (Oregon Health & Science University, USA). For generating OTUB1‐overexpressing cells, plasmids were transfected into HNSCC cells by using Fugene HD (Promega). After transfection, the cells were treated with 500 μg/mL of G418, and then we picked up clones.

For lentiviral gene transfer, CSII‐CMV‐IRES‐Bsd and packaging plasmids (pCAG‐HIVgp and pCMV‐VSV‐G‐RSV‐Rev) were obtained from Dr. Hiroyuki Miyoshi (RIKEN, Japan). Lentiviral vectors expressing Myc‐tagged DUBs were generated by using the In‐Fusion cloning kit (Clontech). Lentiviral vectors and packaging plasmids were transfected into Lenti‐X 293 T cells (Takara Bio Inc.) by using PEI max (Polysciences). Supernatants were collected at 48 h after transfection and filtered using 0.45 μm membrane. Filtered supernatants with 4 μg/mL polybrene were directly added to HSC2 cells. After 24 h, the medium was replaced by fresh media with 5 μg/mL Blasticidin (Thermo Fisher Scientific).

siRNA was transfected with Lipofectamine RNAiMAX (Thermo Fisher Scientific). The human OTUB1 (5′‐GCAAGUUCUUCGAGCACUU‐3′) and human YAP (5′‐GCAACUCCAACCAGCAGCAACAGAU‐3′) siRNAs were obtained from Thermo Fisher Scientific.

### 2.6 Western blotting

Western blotting was performed as described previously.^13^ Cells were lysed using lysis buffer (50 mM pH 7.6 Tris–HCl, 150 mM NaCl, 1 mM EDTA, 1.5 mM MgCl_2_, 0.5% Nonidet P‐40, and 10% glycerol) with protease inhibitor cocktail (Nacalai tesque). After centrifugation, the supernatant was collected. Nuclear and cytoplasmic fraction were obtained by using Nuclear Extraction Kit (ab113474, abcam). Protein concentration was measured using the Thermo Fisher BSA protein assay reagent (Thermo Fisher Scientific) by measuring absorbance at 562 nm with a microplate reader (TECAN infinite 200Pro). Lysates were electrophoresed on 5%–20% gradient polyacrylamide gel (ATTO Corporation) and subsequently transferred to a nitrocellulose membrane (GE Healthcare Life Science). The signal was detected by an Amersham ImageQuant 800 (GE Healthcare Life Science) using a Western ECL Substrate (BIO‐RAD).

### 2.7 In vitro invasion assay

Invasion ability was determined by an in vitro invasion assay using an 8 μm pore cell culture insert (Corning Life Sciences) in a 24‐well plate. Twenty μg of Matrigel (Corning Life Sciences) were coated on the filter as a reconstituted basement membrane substance. Five hundred μL of complete medium was added to the lower compartment. 1.5 × 10^5^ cells were plated on the upper compartment of the cell culture insert with 100 μL of complete medium. After incubation for 24 h, the cells were fixed with formalin and stained with hematoxylin. By wiping with a cotton swab, the cells on the upper surface of the filter were removed, and the number of invaded cells on the lower surface was counted under a light microscope at 100× magnification. The assay was conducted three times, and randomly selected three fields on the filter were counted.

### 2.8 Tumorigenesis assay

Eight‐week‐old male athymic (nude) mice were purchased from Charles River Laboratories. The nude mice (*n* = 5 for each group) were orthotopically injected with 5.0 × 10^5^ HNSCC cells in 100 μL PBS into tongue. After 3–4 weeks, all mice were sacrificed. Tumor volume was calculated by volume = length×(width^2^)/2. Organs, including the tongue, were resected from the mice and fixed in 10% phosphate‐buffered formaldehyde (pH 7.2). Histological examination was performed using HE‐stained sections. All experiments were performed after the administration of anesthesia, and all efforts were made to minimize suffering.

### 2.9 Immunofluorescence

Cells were grown on a 96 well plate. By using BD Cytofix/Cytoperm™ Fixation/Permeabilization Solution Kit (BD biosciences), cells were fixed in fixation buffer for 30 min and were permeabilized with permeabilization/wash buffer. After washing by PBS, the cells were incubated with the primary antibodies (anti‐YAP, anti‐OTUB1, and anti‐FLAG antibodies). As a secondary antibody, Alexa Fluor 488‐conjugated antimouse or rabbit IgG and Alexa Fluor 594‐conjugated antimouse or rabbit IgG were used (Invitrogen). DNA was visualized by hoechst33342 staining. Immunostaining of the cell preparations was recorded by using an epifluorescence Zeiss Axioplan 2 microscope (Zeiss, Inc.) attached to a charge‐coupled‐device camera.

### 2.10 YAP score calculation

Cordenonsi_YAP_conserved gene list (M2871) was obtained from MSigDB (https://www.gsea‐msigdb.org/gsea/msigdb). The microarray data (GSE66949) from HNSCC cell line (SCC‐2) treated with siRNA for *YAP1* (siYAP) was analyzed using the *GEO2R* web tool, and a list of differentially expressed genes between control siRNA and siYAP‐treated cells was obtained. The log‐transformed TPM values of 60 HNSCC cell lines (DepMap 23Q2 files) were obtained from the DepMap portal website (https://depmap.org/portal/). The scores of 19 overlapping genes between the YAP signature (M2871) and the downregulated genes by siYAP treatment (GSE66949) in the 60 HNSCC cell lines were calculated using ssGSEA with the *ConsensusTME* R package.

### 2.11 Statistical analysis

For Kaplan–Meier overall survival, the log‐rank test was used for comparing two groups. For cell proliferation assay, the ANOVA test was used for comparing groups. For in vitro invasion assay, qPCR analysis, and percentage of YAP positive cells, the *t*‐test was used.

## 3 RESULTS

### 3.1 Screening of DUBs associated with HNSCC progression

**FIGURE 1:**
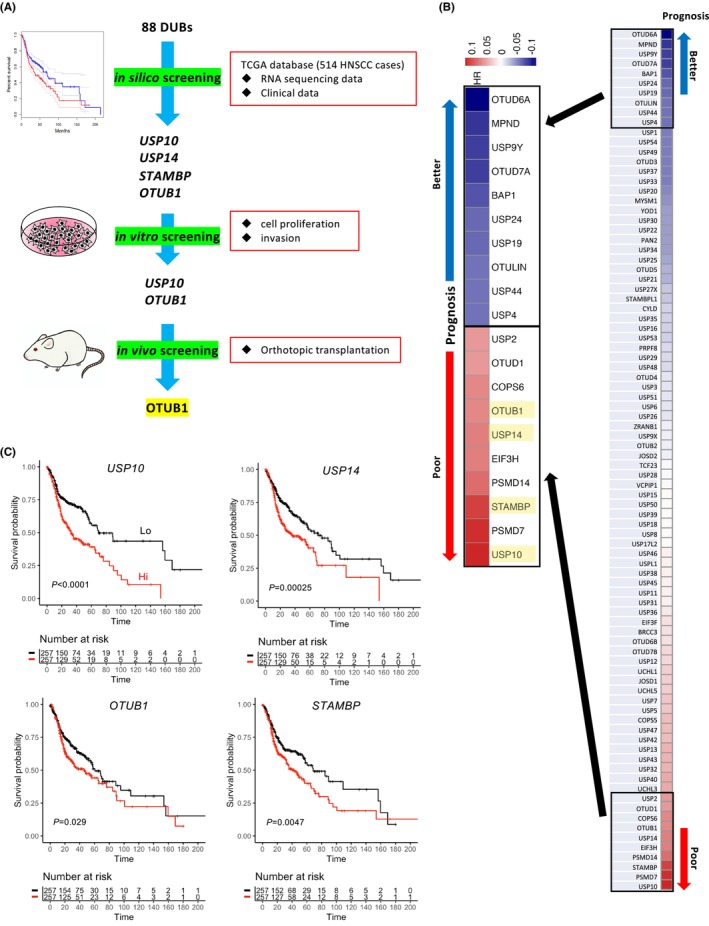
Identifying DUBs involved in the malignant behaviors of HNSCC. (A) The strategy for identifying DUBs involved in the malignant behaviors of HNSCC. (B) The log10 transformed Hazard ratios (HR) of DUBs were visualized as a heatmap in the upper panel. DUBs with the top 10 and bottom 10 hazard ratios (HR) were selected. (C) The patients with higher median expression levels of the selected four DUBs showed poor overall survival.

To identify DUBs correlated with poor prognosis of HNSCC patients, our initial screening process involved analyzing RNA sequencing data and clinical information of 88 DUBs sourced from the TCGA database (Figure 1A). Through this screening, we identified USP10, USP14, OTUB1, and STAMBP as potential candidates (Figure 1A–C). Given that PSMD7 and PSMD14 function as proteasome subunits, and EIF3H serves as a component of the eukaryotic translation initiation factor, they were excluded from consideration as DUBs in this context.

To further elucidate the effects of overexpressing USP10, USP14, OTUB1, and STAMBP in HNSCC cells, we assessed the expression of these DUBs across 11 HNSCC cell lines using qPCR. Among these cells, HSC2 cells exhibited notably lower expression levels of all four DUBs (Figure S1A). Subsequently, HSC2 cells were engineered to overexpress Myc‐tagged DUBs. Validation of the overexpression of these DUBs was confirmed at both mRNA (Figure S1B) and protein levels (Figure S1C).

**FIGURE 2:**
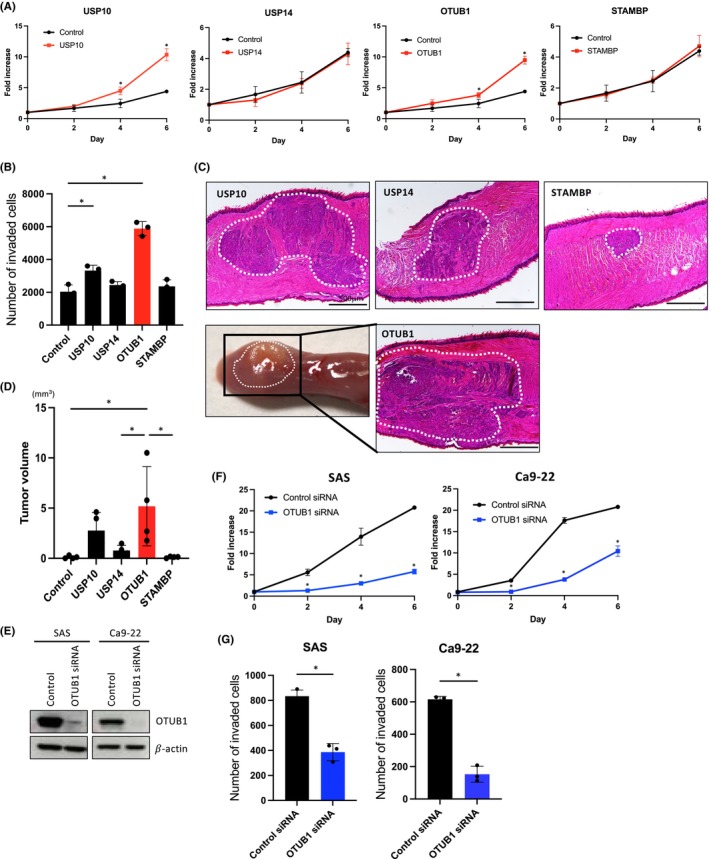
Enhanced invasive and proliferative abilities by OTUB1. (A) Cell proliferation of USP10, USP14, OTUB1, and STAMBP‐overexpressing HSC2 cells. Cell numbers were counted at 0, 2, 4, and 6 days. *p value <0.05. (B) Invasion ability of USP10, USP14, OTUB1, and STAMBP‐overexpressing HSC2 cells. Graph shows the number of invaded cells. Three independent experiments were used to calculate the mean ± S.D. *p value <0.05. (C) In vivo tumorigenesis assay. The representative HE‐stained images of histopathologic sections from mice injected with USP10, USP14, OTUB1, and STAMBP‐overexpressing HSC2 cells into tongue. Scale bar: 500 μm. A gross appearance of the tongue tumor mass from mice injected with OTUB1‐overexpressing HSC2 cells is shown. (D) Graph shows the tumor volume (mm^3^). The experiments were performed with five mice assigned to each group. *p value <0.05. (E) OTUB1 knockdown in SAS and Ca9‐22 cells. OTUB1 siRNA was transfected into HNSCC cells. OTUB1 expression was examined by Western blotting. 𝛽‐Actin expression was used as a loading control. (F) Cell proliferation of OTUB1 siRNA‐treated SAS and Ca9‐22 cells. Cells were counted at 0, 2, 4, and 6 days. *p value <0.05. (G) Invasion ability of OTUB1 siRNA‐treated SAS and Ca9‐22 cells was assessed by in vitro invasion assay. Graph shows the number of invaded cells. Three independent experiments were used to calculate the mean ± S.D. *p value <0.05.

To probe into the functional roles of USP10, USP14, OTUB1, and STAMBP in HSC2 cells, we conducted assays to measure cell proliferation, in vitro invasion, and in vivo tumor growth. Among these DUBs, OTUB1, and USP10 significantly enhanced cell proliferation and invasion (Figure 2A,B, Figure S2A). Notably, OTUB1 overexpression led to the largest tumors in the tongues by orthotopically transplantation into the tongues of nude mice (Figure 2C,D). Noteworthy, only OTUB1 overexpression resulted in cervical lymph node and lung metastasis (Figure S2B,C). Hence, OTUB1 demonstrated the most aggressive phenotypes both in vitro and in vivo within HNSCC cells.

### 3.2 Promotion of cell proliferation and invasion by OTUB1 in HNSCC cells

To bolster the confirmation of the functional role of OTUB1, we delved into the proliferative and invasive capabilities of HNSCC with manipulated OTUB1 expression levels—both overexpression and knockdown. We induced the expression of FLAG‐tagged OTUB1 in HSC2 and HSC3 cells with lower endogenous levels of OTUB1 (Figure S3A). We confirmed that OTUB1 overexpression led to a significant augmentation in both cell proliferation and invasion (Figure S3B,C). Conversely, we employed siRNA to deplete OTUB1 in SAS and Ca9‐22 cells, where the baseline expression levels of endogenous OTUB1 were comparatively higher (Figure 2E). OTUB1 depletion brought about a substantial reduction in cell proliferation and invasion within both cells (Figure 2F,G). These findings serve to further solidify the concept that OTUB1 assumes a pivotal role in driving cell proliferation and invasion in HNSCC cells.

### 3.3 Regulating YAP1 through OTUB1 in HNSCC progression

**FIGURE 3:**
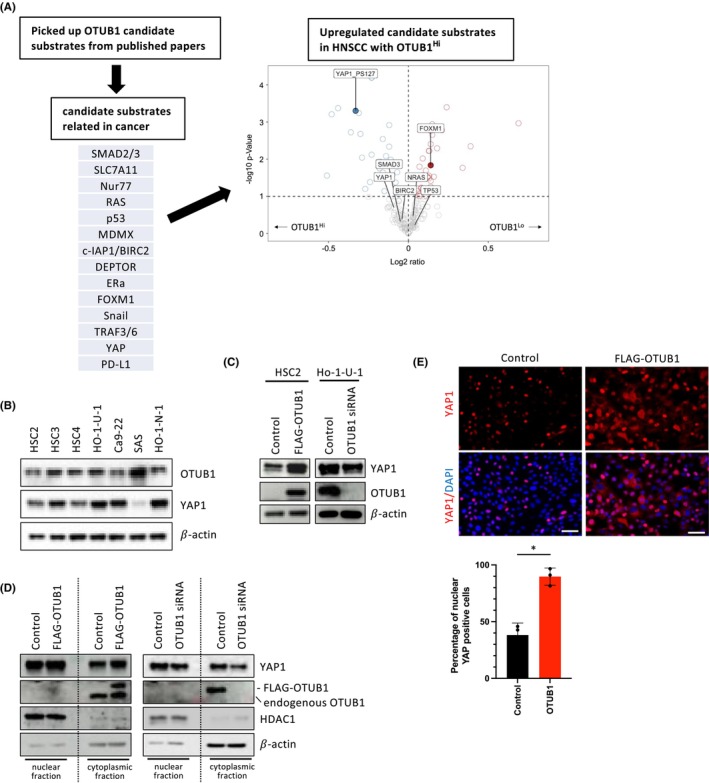
Regulating YAP1 by OTUB1 in HNSCC progression. (A) Candidate substrates for OTUB1 were initially chosen based on published papers, further refined by focusing on those related to cancer, and ultimately narrowed down to substrates that exhibited upregulation in HNSCC with high OTUB1 expression. The Volcano plot illustrates the differences in protein expression between the groups of HNSC patients with median‐high and median‐low OTUB1 mRNA expression. The genes with higher expression in OTUB1‐high and OTUB1‐low groups are illustrated as blue circles and red circles each. The genes which reported as substrates of OTUB1 are highlighted in the Volcano plot. (B) Protein expression levels of OTUB1 and YAP1 in HNSCC cell lines by immunoblotting with indicated antibodies. 𝛽‐Actin expression was used as a loading control. (C) Protein expression levels of YAP1 and OTUB1 in OTUB1‐overexpressing HSC2 cells and OTUB1 siRNA‐treated Ho‐1‐U‐1 cells by immunoblotting with indicated antibodies. 𝛽‐Actin expression was used as a loading control. (D) Protein expression levels of YAP1 and OTUB1 in nuclear and cytoplasmic fraction of OTUB1‐overexpressing HSC2 cells by immunoblotting with indicated antibodies. HDAC expression was used as a loading control of nuclear fraction, and 𝛽‐Actin was used as a loading control of cytoplasmic fraction. (E) The representative immunofluorescence images of YAP1 expression in OTUB1‐overexpressing HSC2 cells are shown in upper panel. Nuclei were counterstained with DAPI. Graph shows the percentage of YAP1 positive cells (lower panel). Three independent experiments were used to calculate the mean ± S.D. *p value <0.05.

In previous studies, xCT/SLC7A11,^14^ FOXM1,^15^, ^16^, ^17^ Snail,^18^ PD‐L1,^19^ YAP1,^20^ SMAD2/3,^21^ Nur77,^22^ RAS,^23^ p53,^24^ MDMX,^25^ c‐IAP1/BIRC2,^26^ DEPTOR,^27^ Era,^28^ and TRAF3/6^29^ were identified as OTUB1 substrates in cancer progression (Table S1). Among them, we explored upregulated substrates at protein levels in HNSCC cases with high OTUB1expression by using the “TCGA‐HNSC” protein expression datasets obtained from the cBioPortal (Figure 3A). We divided HNSCC patients into median high and low OTUB1 mRNA expression groups and compared the protein expression between these groups. In high OTUB1 mRNA expression group, YAP was upregulated at protein levels (Figure 3A). Consequently, we selected YAP1 as a substrate for further in‐depth analysis of OTUB1 involvement.

In HNSCC cells, a robust correlation between the expression of OTUB1 and YAP1 was observed (Figure 3B and Figure S4A). Moreover, YAP1 exhibited a substantial increase, while the other substrates remained unchanged in OTUB1‐overexpressing cells (Figure 3C and Figure S4B). Conversely, OTUB1 depletion resulted in decreased levels of YAP1 (Figure 3C and Figure S4B). Furthermore, OTUB1 overexpression or depletion influenced on both nuclear and cytoplasmic YAP1 expression (Figure 3D). Immunofluorescence analysis revealed that nuclear YAP1‐positive cells were significantly increased by OTUB1 overexpression (Figure 3E). These findings suggest that OTUB1 may activate YAP1 transcriptional activity via its protein stabilization.

**FIGURE 4:**
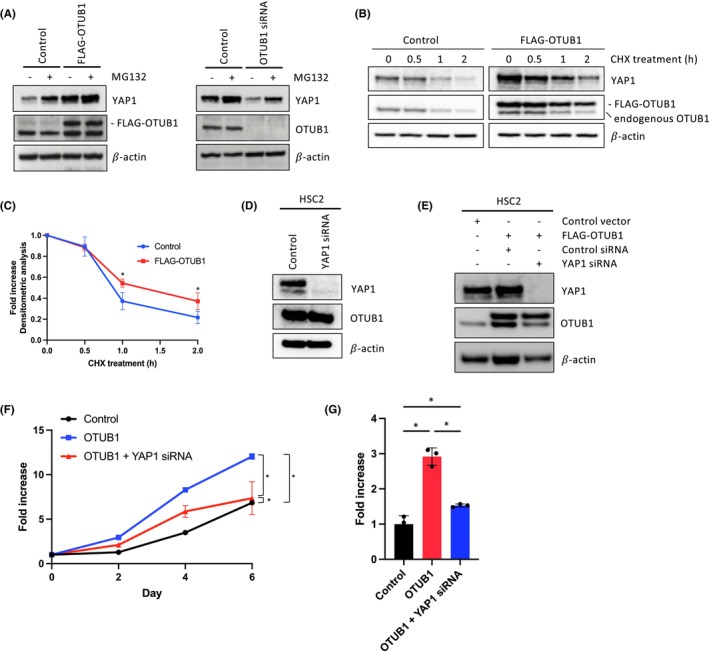
Impact of YAP1 on the OTUB1‐driven invasion and proliferation of HNSCC cells. (A) Protein expression levels of YAP after 8 h of MG132 treatment (10 μM) in OTUB1‐overexpressing HSC2 cells or OTUB1 siRNA‐treated Ho‐1‐U‐1 cells by Western blotting. Ectopic and endogenous OTUB1 expression were confirmed by Western blotting. 𝛽‐Actin expression was used as a loading control. (B) Protein expression levels of YAP after CHX treatment (50 μg/mL) at indicated time in OTUB1‐overexpressing HSC2 cells by Western blotting. Ectopic and endogenous OTUB1 expression were confirmed by Western blotting. 𝛽‐Actin expression was used as a loading control. (C) Densitometric analysis of YAP1 protein expression levels after CHX treatment in control and OTUB1‐overexpressing HSC2 cells. Three independent experiments were used to calculate the mean ± S.D. *p value <0.05. (D) YAP1 was depleted by siRNA in HSC2 cells. Protein expression levels of YAP1 and OTUB1 by Western blotting. 𝛽‐Actin expression was used as a loading control. (E) YAP1 was depleted by siRNA in OTUB1‐overexpressing HSC2 cells. Protein expression of YAP1 and OTUB1 was determined by Western blotting. 𝛽‐Actin expression was used as a loading control. (F) Cell proliferation of OTUB1‐overexpressing HSC2 cells with or without YAP1 knockdown. Cells were counted at 0, 2, 4, and 6 days. *p value <0.05. (G) Invasion ability of OTUB1‐overexpressing HSC2 cells with or without YAP1 knockdown was assessed by in vitro invasion assay. Graph shows the number of invaded cells. Three independent experiments were used to calculate the mean ± S.D. *p value <0.05.

To delve into the regulatory effect of OTUB1 on the stability of YAP1 protein in HNSCC cells, we conducted additional investigations. Initially, we examined the impact of OTUB1 on the accumulation of YAP1 protein by treating cells with the proteasome inhibitor. MG132 treatment led to an elevation in YAP1 protein levels in control cells, while the impact of MG132 on YAP1 protein levels induced by OTUB1 overexpression was relatively minimal (Figure 4A). Furthermore, MG132 treatment counteracted the reduction in YAP1 protein levels resulting from OTUB1 depletion (Figure 4A). Additionally, we further confirmed the impact of OTUB1 on the stability of YAP1 protein using CHX treatment, which inhibits protein synthesis. OTUB1 overexpression resulted in an increased half‐life of YAP1 protein (Figure 4B,C). These findings suggest that OTUB1 might modulate YAP1 protein levels through its involvement in the ubiquitin‐proteasome pathway, thus regulating YAP1 stability.

To advance our understanding of the role of YAP1 in the phenotypes driven by OTUB1 in HNSCC cells, we assessed the efficacy of YAP1 siRNA in mitigating the cell proliferation and invasion prompted. YAP1 siRNA treatment led to a significant decrease in YAP1 protein levels (Figure 4D). Subsequently, we depleted YAP1 in OTUB1‐overexpressing HSC2 cells (Figure 4E). Notably, YAP1 depletion rescued the cell proliferation and invasion that were induced by OTUB1 overexpression (Figure 4F,G).

### 3.4 A positive correlation between OTUB1 expression and YAP signaling

**FIGURE 5:**
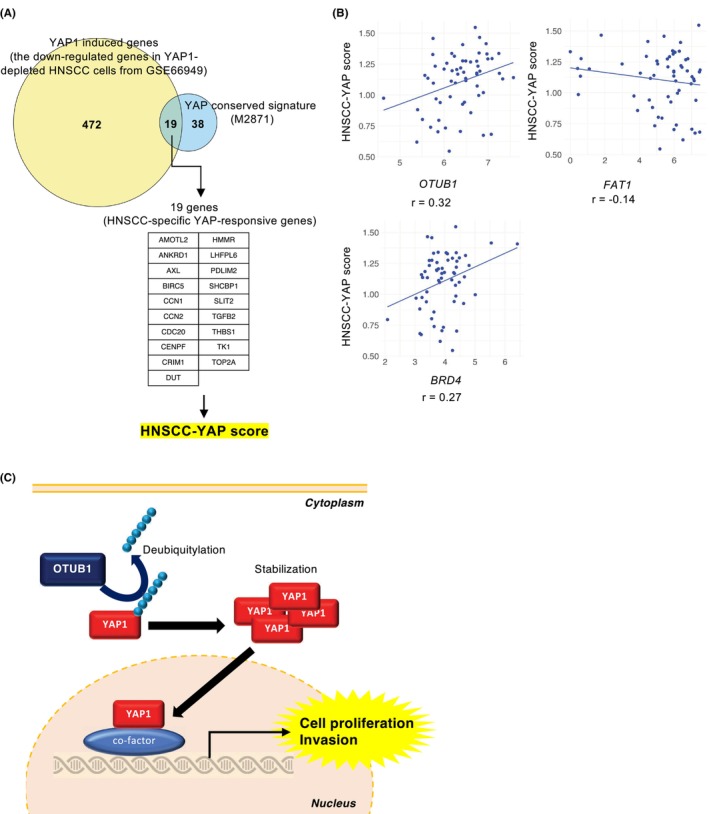
Correlation between OTUB1 expression and YAP responsive genes. (A) Venn diagram shows the number of overlapping genes between the YAP conserved signature and the downregulated genes after siYAP1 treatment (YAP induced genes) in SCC‐2 cells. The scores of HNSCC‐specific YAP responsive genes were calculated from the overlapping 19 genes in 60 HNSCC cell lines. (B) The correlations between the YAP score and the expression level of *OTUB1*, *FAT1*, and *BRD4* were shown in the scatter plots. Spearman's correlation coefficients were described in the plot.

We embarked on a comprehensive analysis to discern the intricate relationship between OTUB1 and YAP signaling pathway. In our quest to pinpoint genes particularly responsive to YAP in HNSCC, we juxtaposed the YAP conserved signature available from MSigDB with genes downregulated in YAP1‐depleted HNSCC cells, sourced from GSE66949. Through this analysis, we identified a set of 19 genes that exhibited an overlap, constituting what we term as “HNSCC‐specific YAP‐responsive genes”. Subsequently, we evaluated the expression of these genes across 60 HNSCC cell lines and derived “HNSCC‐YAP score” indicative of their expression levels (Figure 5A).

Continuing our exploration, we examined the correlations existing between the HNSCC‐YAP score and the expression profiles of *OTUB1*, *FAT1*, and *BRD4* in HNSCC cells. FAT1, an upstream inhibitor of YAP1, frequently undergoes mutations in HNSCC via missense or truncating mutations.^30^ Additionally, the YAP/TAZ transcriptional complex triggers an active chromatin state by recruiting BRD4 to deposit active histone marks in FAT1‐mutant HNSCC cells.^30^ Our analysis revealed an inverse correlation between *FAT1* and the HNSCC‐YAP score, while a positive correlation was observed with *BRD4* (Figure 5B). Of particular interest, both *OTUB1* and *BRD4* displayed a positive correlation with the HNSCC‐YAP score (Figure 5B).

## 4 DISCUSSION

In this study, we have identified OTUB1 as a key instigator of HNSCC progression through in silico, in vitro, and in vivo screening of all DUBs. This is the first study to screen DUBs for identifying a potential target for HNSCC treatment. This finding aligns with previous reports highlighting the important role of OTUB1 in various cancer types.^31^, ^32^, ^33^ Furthermore, YAP1 was identified as a substrate of OTUB1 in HNSCC progression. Although YAP1 has already been identified as a substrate of OTUB1 in the regulation of stemness and progression of gastric cancer cells,^20^ there have been no reports on the regulation of YAP1 by OTUB1 in HNSCC to date. Indeed, YAP1 protein levels exhibit an increase in precancerous and invasive HNSCC,^34^ and the striking observation of endogenous YAP1 hyperactivation in tongue tissues leading to swift cancer development strongly implies the role of YAP1 in triggering the onset and progression of HNSCC.^35^ These findings suggest that OTUB1‐YAP1 axis may play an important role in HNSCC progression. It is noteworthy that the significance of both OTUB1 and YAP has been underscored in lung, gastric, and breast cancers.^20^, ^36^, ^37^

The Hippo signaling pathway serves as a crucial cellular signaling pathway that governs a wide range of biological processes.^38^ In an active state of the Hippo pathway, the LATS1/2 kinase phosphorylates and inhibits YAP, leading to its sequestration in the cytoplasm.^39^ Cytoplasmic retention of YAP prevents its nuclear translocation and interaction with transcription factors, thereby suppressing gene transcription.^40^ YAP activation elicits diverse cellular responses, including cell proliferation, cell survival, stem cell self‐renewal, and differentiation. Dysregulated YAP activity has been strongly associated with the development of cancer.^38^, ^41^ Elevated YAP expression has been observed in oral, breast, lung, and liver cancers.^42^, ^43^, ^44^, ^45^ In HNSCC, frequent deletion or truncation of FAT1 disperses Hippo components and leads to aberrant activation of YAP via suppressing its ubiquitination.^46^ As gene alterations of *FAT1* is frequently occurred in HNSCC,^47^ *FAT1* gene alterations may be involved in the prevalent YAP activation in HNSCC. Our present findings propose that OTUB1 may synergistically activate YAP1 with *FAT1* gene alterations in HNSCC. Based on these findings, inhibiting OTUB1 expression or promoting the ubiquitination of YAP1 protein could be potential strategies for therapeutic intervention in HNSCC. This provides a novel direction for the development of targeted drugs that disrupt the interaction between OTUB1 and YAP, offering potential breakthroughs in HNSCC treatment. However, further studies were warranted to elucidate the regulatory network involving OTUB1 and YAP1 in HNSCC cells. Since YAP functions as a transcription factor, a meticulous dissection of its roles in orchestrating other genes and signaling pathways becomes paramount. In this study, our selection of 19 genes for the evaluation of HNSCC‐YAP score adds an interesting dimension (Figure 5A). Notably, a robust correlation between OTUB1 expression and the HNSCC‐YAP score was observed (Figure 5B). It would be intriguing to explore the correlations between OTUB1 and the HNSCC‐YAP score within the context of HNSCC cell phenotypes. Such endeavors promise to contribute to a more holistic comprehension of the operative mechanisms steering the roles of OTUB1 and YAP1 in the evolution of HNSCC.

Here we identified OTUB1 as the most aggressive characteristics among all DUBs in HNSCC. The screening of DUBs in the present study is unique and this study represents the first report on the role of the OTUB1‐YAP1 axis in HNSCC progression (Figure 5C). While we demonstrated a strong correlation between OTUB1 expression and the HNSCC‐YAP score through bioinformatic analysis, we acknowledge a limitation in our ability to access the correlation between OTUB1 expression and nuclear expression of YAP1 in HNSCC tissues. Future research would benefit from addressing this limitation through immunohistochemical analysis using a large scale of HNSCC cases. Our present findings provide valuable insights into the molecular mechanisms underlying HNSCC and present potential avenues for the development of innovative treatment approaches and therapeutic targets.

## AUTHOR CONTRIBUTIONS

**Shengjian Jin:** Data curation (equal); investigation (equal); writing – original draft (equal). **Takaaki Tsunematsu:** Conceptualization (equal); investigation (equal); writing – original draft (equal). **Taigo Horiguchi:** Investigation (equal); supervision (equal). **Yasuhiro Mouri:** Data curation (equal); investigation (equal); supervision (equal). **Wenhua Shao:** Investigation (equal). **Keiko Miyoshi:** Investigation (equal). **Hiroko Hagita:** Investigation (equal). **Motoharu Sarubo:** Investigation (equal). **Natsumi Fujiwara:** Investigation (equal). **Guangying Qi:** Supervision (equal). **Naozumi Ishimaru:** Supervision (equal). **Yasusei Kudo:** Conceptualization (lead); writing – original draft (lead).

## FUNDING INFORMATION

This work was supported by grants to Y. Kudo from a Grant‐in‐Aid for Scientific Research (KAKENHI) from the Ministry of Education, Culture, Sports, Science, and Technology, Japan (22K19629, 22H03288, and 21KK0162).

## CONFLICT OF INTEREST STATEMENT

The authors declare no conflict of interest.

## ETHICS STATEMENT

All animal experiments were approved by the Committee on Animal Experiments of Tokushima University (approval no. T‐2022‐23) and complied with national guidelines, Tokushima University regulations, and institutional by laws. Murine in vivo studies were performed in accordance with the Guidelines for Proper Conduct of Animal Experiments, published by the Science Council of Japan.

## Supporting information


Appendix S1

## Data Availability

Data can be made available upon reasonable request.
