# Supplementary material for: Involvement of the OTUB1‐YAP1 axis in driving malignant behaviors of head and neck squamous cell carcinoma
Source: Cancer Med. 2023 Nov 20;12(24):22156–69. doi: 10.1002/cam4.6735 (PMC10757095; doi:10.1002/cam4.6735)

Supplementary Table S1: List of OTUB1 substrates involved in cancer progression.

Substrate	Cancer Type	Function	Reference No.
SLC7A11	non-specific	CD44-mediated inhibitory effects on ferroptosis	14
FOXM1	Ovarian	Increased proliferation, migration, invasion	15
	Breast	Increased proliferation, epirubicin resistance	16
	Renal cell	Increased proliferation, invasion via ECT2-Rho signaling	17
Snail	Esophageal	EMT, increased migration, invasion	18
PD-L1	Breast	promotes cancer cell immunosuppression	19
YAP	Gastric	Increased proliferation, invasion via Hippo signaling	20
SMAD2/3	non-specific	Increased migration via TGFβ signaling pathway	21
Nur77	non-specific	anti-inflammatory effect	22
RAS	Lung	Increased growth via RAS/MAPK pathway	23
p53	non-specific	Increased apoptosis	24
MDMX	non-specific	Increased apoptosis	25
c-IAP1	non-specific	Decreased apoptosis via NF-κB & MAPK signalling pathways	26
DEPTOR	non-specific	Autophagy via mTORC1 signalling	27
ERa	Endometrial	Decreased ERa transcription	28
TRAF3/6	non-specific	anti-viral response	29

Supplementary Figure S1: Generating USP10, USP14, OTUB1, and STAMBP overexpressing HNSCC cells. (A) Expression of *USP10, USP14, OTUB1, and STAMBP* in HNSCC cell lines by qRT-PCR analysis. Data represent the mean ± SD of triplicates. (B) USP10, USP14, OTUB1, and STAMBP were transfected into HSC2 cells. Expression of ectopic DUBs was confirmed by qRT-PCR. (C) Ectopic expression of Myc-tagged DUBs was confirmed by Western blotting. ß-actin expression was used as a loading control.

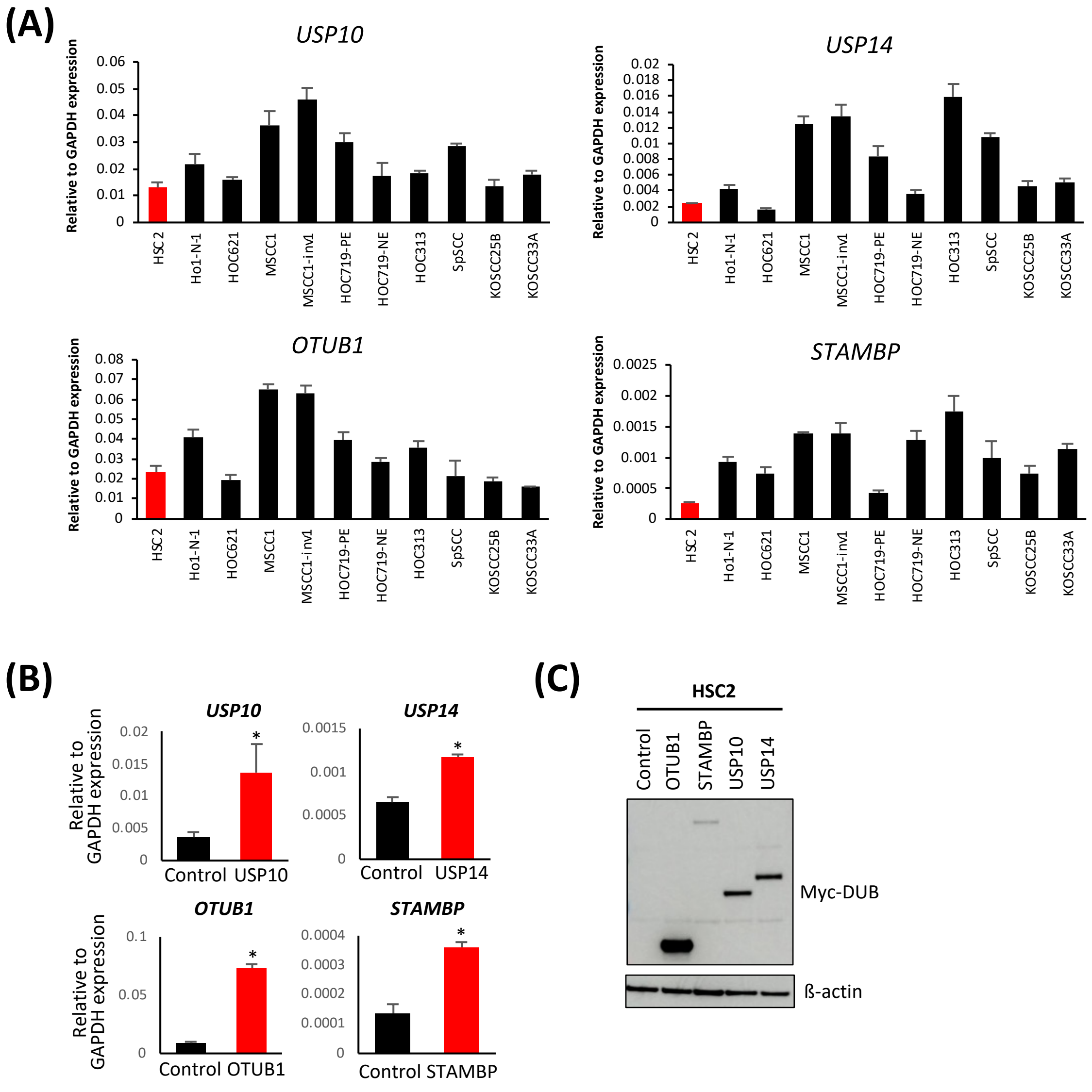

Supplementary Figure S2: Enhanced invasion and metastasis in OTUB1 overexpressing cells. (A) Invasion ability of USP10, USP14, OTUB1, and STAMBP- overexpressing HSC2 cells. The representative images of each group are shown. Scale bar: 100 µm. (B) Summary of the metastasis of USP10, USP14, OTUB1, and STAMBP-overexpressing cells injected mice. Control is empty vector transfected HSC2 cells injected mice. (C) HE-stained images of cervical lymph node metastasis and lung metastasis of OTUB1-overexpressing HSC2 cells.

(A)

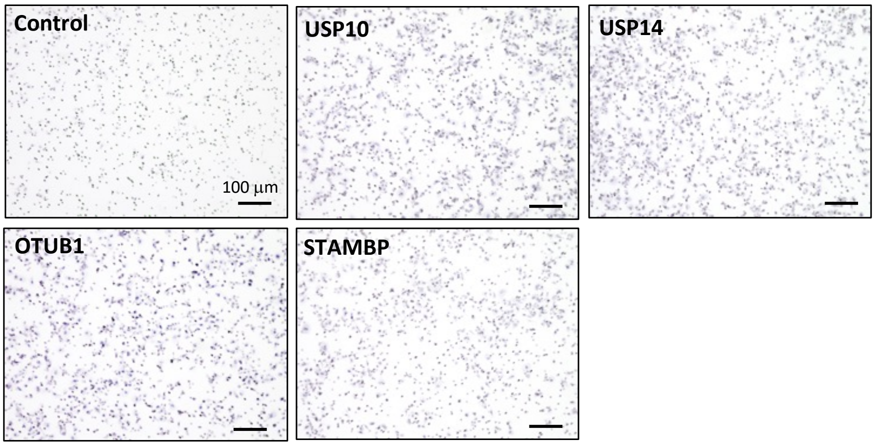

(B)

	N	Tumor formation	Lymph nodes metastasis	Lung metastasis
**Control**	5	2	0	0
**OTUB1**	5	4	1	1
**USP10**	5	4	0	0
**USP14**	5	4	0	0
**STAMBP**	5	3	0	0

(C)

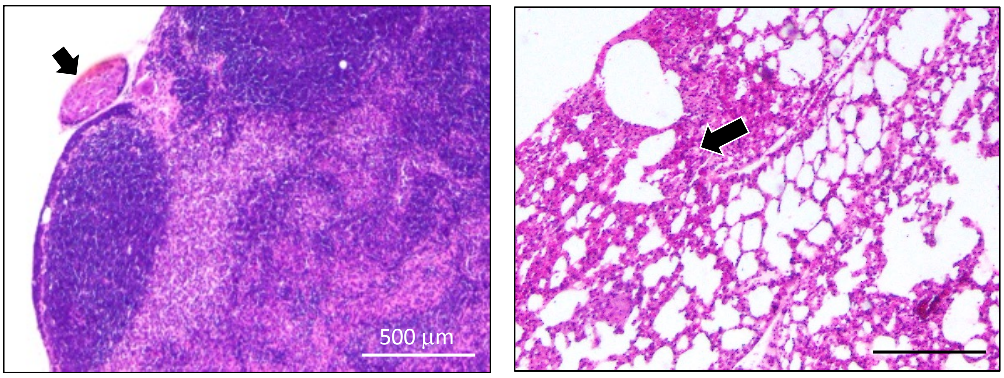

Supplementary Figure S3: Impact of OTUB1 overexpression in cell proliferation and invasion in HNSCC Cells. (A) Generation of OTUB1-overexpressing HSC2 and HSC3 cells. Ectopic OTUB1 expression was examined by immunoblotting with anti-FLAG antibody. β-actin expression was used as a loading control. (B) Cell proliferation of OTUB1-overexpressing HSC2 and HSC3 cells. Cells were counted at 0, 2, 4, and 6 days. *P-value < 0.05. (C) Invasion ability of OTUB1-overexpressing HSC2 and HSC3 cells was assessed by *in vitro* invasion assay. Graph shows the number of invaded cells. Three independent experiments were used to calculate the mean ± S.D. *P-value < 0.05.

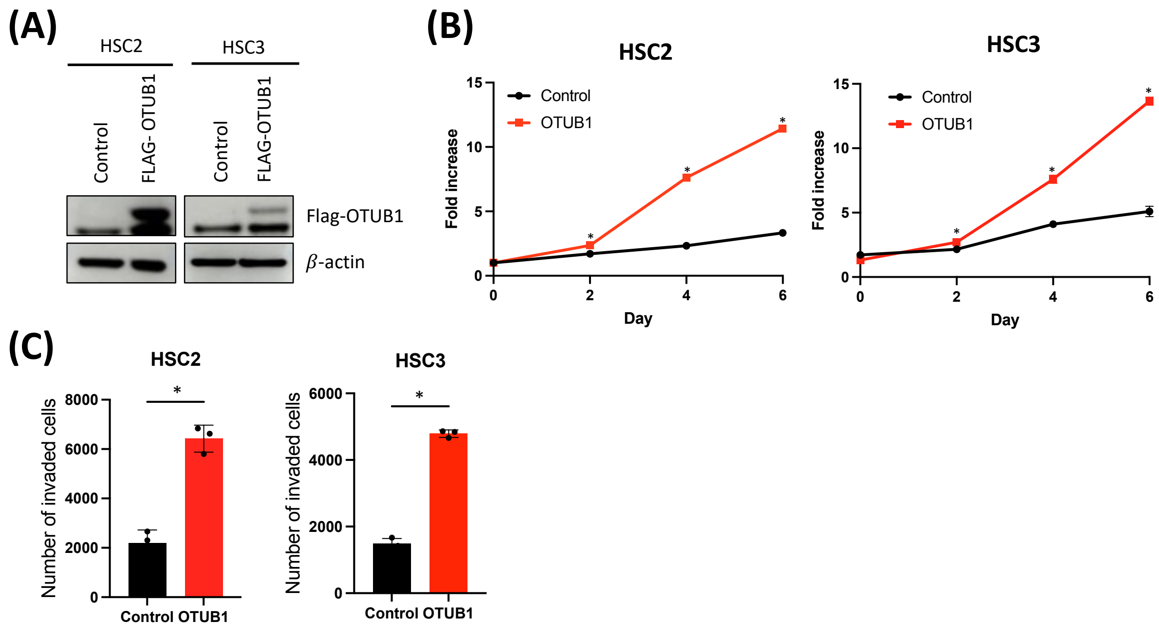

Supplementary Figure S4: Protein expression profiles of OTUB1 substrates in HNSCC cells. (A) Protein expression levels of OTUB1, PD-L1, xCT, FOXM1, and Snail in HNSCC cell lines by immunoblotting with indicated antibodies. β-actin expression was used as a loading control. Using the same extracts as those employed in Figure 3B, we conducted an examination of OTUB1 substrate expression. The images of OTUB1 and ß-actin expression were reused for this analysis. (B) Protein expression levels of PD-L1, xCT, FOXM1, Snail, and OTUB1 in OTUB1-overexpressing HSC2 cells and OTUB1 siRNA treated Ho-1-U-1 cells by immunoblotting with indicated antibodies. β-actin expression was used as a loading control. Using the same extracts as those employed in Figure 3C, we conducted an examination of OTUB1 substrate expression. The images of OTUB1 and ß-actin expression were reused for this analysis. (C) Invasion ability of OTUB1-overexpressing HSC2 cells with or without YAP1 knockdown was assessed by *in vitro* invasion assay. Graph shows the number of invaded cells. Three independent experiments were used to calculate the mean ± S.D. **P*-value < 0.05.

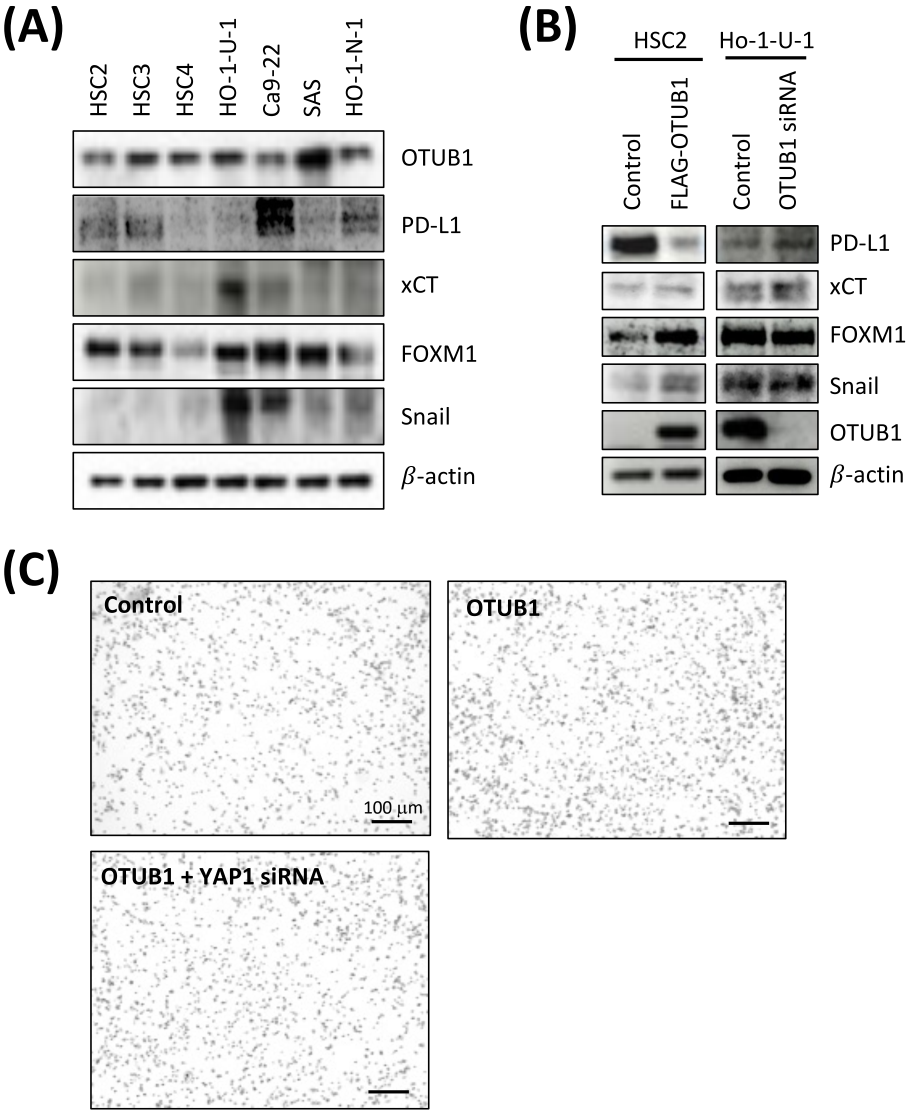

Supplement: Supplementary file 1 — Appendix S1 [file CAM4-12-22156-s001.pdf]
